# Allergic bronchopulmonary aspergillosis: A review of 42 patients from a tertiary care center in India

**DOI:** 10.4103/0970-2113.48895

**Published:** 2009

**Authors:** R. Prasad, R. Garg, Sanjay, A. D. Shukla

**Affiliations:** *Department of Pulmonary Medicine, King GeorgeÊs Medical University, Lucknow-226 003, India*

**Keywords:** Allergic bronchopulmoary aspergillosis, asthma, CT scan, India, outcome

## Abstract

**Objective:**

To study the clinical, radiological, and laboratory profile in patients of allergic bronchopulmonary aspergillosis (ABPA).

**Materials and Methods:**

Retrospective analysis of 42 cases of ABPA, diagnosed over a period of 10 years from 1995 to 2005, for their clinical, radiological, and laboratory profiles.

**Results:**

Of 42 ABPA patients, 27 were men and 17 were women. Their mean age at the time of diagnosis was 31.2 years and mean duration of illness was 12.2 years. Breathlessness was the chief symptom. Other allergic disorders existed in 17 (40.5%) patients, and family history suggestive of allergic disease was present in 22 (52.4%) patients. Most common chest radiographic finding was fleeting pulmonary shadows in 28 (66.7%) patients. High resolution CT thorax revealed central bronchiectasis as predominant finding. Peripheral blood eosinophilia more than 1000 cells/μl, Type I and type III cutaneous reactivity to *Aspergillus* antigen, elevated serum titers of total IgE antibody, *A. fumigatus* specific IgE and IgG antibodies, and serum precipitin against *A. fumigatus* were positive in majority of patients, who underwent these tests. Thirty eight (90.5%) patients had had history of antitubercular treatment during the course of their illness. All 42 patients met at least four criteria for the diagnosis of ABPA.

**Conclusion:**

Any patient of bronchial asthma, presenting with recurrent shadows in chest radiograph and high peripheral blood eosinophilia, should be investigated for ABPA. Efforts need to be intensified to improve the awareness level among general physicians for early diagnosis and prompt treatment of this disease to avoid misuse of antitubercular drugs.

## INTRODUCTION

Allergic bronchopulmonary aspergillosis (ABPA) is most frequently recognized manifestation of pulmonary aspergillosis occurring worldwide, and is also an important emerging disease in India.^1^ Being an immunologically mediated disease, it usually occurs in atopic individuals and is caused by hypersensitivity reaction to fungus *A. fumigatus*. This potentially destructive lung disease was first recognized in England^2^ in 1952 and in India^3^ in 1971, followed by various reports by various authors from across the country^4^–^11^ [Table 1]. However, the disease is still underdiagnosed in India and various reports have documented that 30–48% of ABPA patients are initially misdiagnosed as pulmonary tuberculosis. In the present study, a retrospective analysis of clinical, radiological, and laboratory profiles of 42 patients with ABPA were done.

**Table 1:** Results of the systematic review of published literature from India^5^–^11^

Features	Bedi 1994	Behera 1994	Kumar 2000	Kumar and Gaur 2000	Chakrabarti 2002	Shah 2003	Agarwal 2006	Our study 2007
Total	20	35	29	32	89	113	126	42
Mean age (years)	NA	34.3	36.3	34	36.4	32	34.4	31.2
Mean duration of asthma (years)	NA	11.1	12.5	12	12.1	11	8.8	12.2
History of asthma (%)	100	94	100	100	90	99	100	100
Expectoration of sputum plugs (%)	55	NA	34.4	31	69	37	47.6	38.1
Peripheral eosinophilia (%)	100	43	100	100	100	83	NA	100
Fleeting shadows (%)	85	77	28	69	74	89	36.5	66.7
Skin test to Aspergillus					
Type I (%)	100	80	100	100	82	100	100	97.6
Type III (%)	35	29	44.8	53	26	100	75.4	69.1
Elevated total IgE	NA	NA	100	100	NA	100	NA	100
Aspergillus-specific IgE/IgG	NA	NA	100	100	NA	96	NA	81
Serum precipitins against *Aspergillus* (%)	100	77	79	75	72	85	NA	100
Central bronchiectasis (%)	NA	71	83	77	69	99	73.1	100
History of anti tuberculous drugs (%)	NA	34	NA	NA	29	81	46.8	91

NA: Not available

## MATERIALS AND METHODS

The study is a retrospective analysis of 42 patients with ABPA, diagnosed over a period of 10 years from 1995–2005, at Department of Pulmonary Medicine, King George's Medical University, Lucknow. Diagnosis of ABPA was considered if they met four of the following major criteria: (1) history of asthma; (2) radiographic pulmonary infiltrates (fixed/transient); (3) central bronchiectasis on HRCT thorax; (4) elevated total serum IgE antibody; (5) Type I cutaneous reactivity to *Aspergillus* antigen; (6) precipitating antibodies to *A. fumigatus;* (7) elevated specific serum IgE and IgG to *A. fumigatus*; and (8) peripheral blood eosinophilia.

These ABPA patients were analyzed retrospectively for their clinical, radiological, and laboratory profiles. Clinical evaluation was done by detailed history of present illness, presence of other allergic disorders, history of past treatment, family history of atopic disorder, and physical examination. Radiological examination was done by plain chest radiograph and high resolution computed tomography of thorax. Laboratory profile was analyzed by peripheral blood absolute eosinophil count, skin testing to *Aspergillus* antigen, serum titers of total IgE antibody, *A. fumigatus* specific IgE and IgG antibodies, and serum precipitin against *A. fumigatus*.

## RESULTS

### Demographic characteristics

A total of 42 cases of ABPA were diagnosed in our hospital from 1995–2005, including 27 male and 15 female patients. Out of these 42 patients, 29 patients (69%) were in the age group of 20–40 years. In our study group the youngest patient was 9-years old and the oldest was 60 at the time of diagnosis. The mean age was 31.2 years.

### General features of patients

Mean duration of their illness was 12.2 years (1.5–30 years). Data for stage of disease were available for 19 patients, which showed that three (15.8%) patients were in stage I; one (5.3%) patient in stage II; ten (52.6%) in stage III; one (5.3%) in stage IV; and one (5.3%) in stage V. Symptom profile of these patients revealed that all 42 (100%) patients had complaint of breathlessness; 24 (57.1%) had cough; 16 (38.1%) were passing mucus plugs in sputum; 11 (26.2%) had fever; 12 (28.6%) had hemoptysis; and 4 (9.5%) had chest pain. History of other allergic disorders, like allergic rhinitis and dermatitis was present in 17 (40.5%) patients, and family history suggestive of allergic diseases was present in 22 (52.4%) patients. Out of 42 patients, 34 (81%) patients frequently needed corticosteroids for control of their symptoms. Past history of antitubercular treatment was present in 38 (90.5%) patients, sometime during the course of their illness.

### Radiological pattern

Radiological examination of these patients revealed normal chest radiographs in only two (4.8%) patients. Among the other 40 patients with abnormal chest radiographs, fleeting pulmonary infiltrates were the most common finding and distribution of shadows were mainly in upper zones. Fleeting shadows were present in 28 (66.7%) patients and fixed shadows were seen in remaining 12 (28.6%) patients. Shadows seen were, infiltrates in 29 (69.1%) patients; consolidation in 8 (19.1%); ring shadows in 4 (9.5%); gloved finger appearance in 2 (4.8%); tram track appearance in 1 (2.4%); and prominent bronchovascular markings in 7 (16.7%) patients. Distribution of shadows was unilateral in 14 (33.3%) patients and bilateral in 26 (61.9%) patients. They were in upper zone in 29 (69%) patients and in lower zone in 21 (50%) patients. HRCT thorax showed central bronchiectasis in all 14 patients (100%), who were able to afford it. One patient had cavitation, with aspergilloma, and three patients showed air bronchogram, suggestive of consolidation.

### Laboratory profile

All patients underwent tests like peripheral blood absolute eosinophil count and skin test for Type I and Type III cutaneous reactivity to *Aspergillus* antigen. Peripheral blood absolute eosinophil count was raised in all and in 37 (88.1%) patients it was >1000 cells/*μ*l. Skin test for Type I cutaneous reactivity was positive in 41 (97.6%) patients, while Type III reaction was positive in 29 (69.1%) patients. Sputum culture for fungal elements revealed growth of *A. fumigatus* in 27/39 (69.2%) patients. Serum titers for specific IgE/IgG were elevated in 30/37 (81.1%) patients. Total serum IgE titers were elevated in all 16 patients who underwent this test (100%). Serum precipitin test against *A. fumigatus*, done in 18 patients, was positive in all. All patients were not subjected to all laboratory tests, either due to financial constraints or lack of consent. Only 14 patients were subjected to all tests and out of 14 patients, 13 (93%) m*e*t all the criteria of ABPA. All 42 patients met at least four criteria for the diagnosis of ABPA [Table 2].

**Table 2:** Allergic bronchopulmonary aspergillosis diagnostic criteria

Diagnostic criteria	Number of patients* (%)
Asthma	42 (100)
Type I skin test to Aspergillus	41 (97.6)
Peripheral eosinophilia	42 (100)
Radiographic pulmonary infiltrates	40 (95.2)
Central bronchiectasis on HRCT thorax	14 (33.3)
Elevated total serum IgE	16 (38.1)
Precipitating antibodies to Aspergillus	18 (42.9)
Aspergillus specific IgE and IgG	30 (71.4)
All eight criteria	13 (31)
Four criteria	42 (100)

Total number of patients, N = 42

## DISCUSSION

ABPA is a syndrome characterized by bronchial asthma, recurrent chest radiographic infiltrates, peripheral blood eosinophilia, frequent need of corticosteroid therapy for control of symptoms, and significant lung destruction. Despite several published series from various parts of the country, this disease is still underrecognized and misdiagnosed as pulmonary tuberculosis.^5^–^11^ This has serious clinical implications as patients with ABPA often receive antitubercular therapy for a long time while lung damage continues to progress relentlessly. In our study, 91% of patients had been misdiagnosed as pulmonary tuberculosis, and they had been treated with antitubercular therapy before a diagnosis of ABPA could be made. Recent studies^8^,^11^ have also showed that 81 and 46.8% of ABPA patients were misdiagnosed as pulmonary tuberculosis in Delhi and Chandigarh, respectively. This is due to lack of awareness among general physicians or lack of diagnostic facilities for ABPA. To prevent chronic lung damage, a high level of suspicion is necessary so that correct diagnosis can be made at an early stage. The clinical profile of our patients was similar to the observations reported by previous investigators.^5^–^11^

Oral corticosteroids remain the cornerstone for treatment of ABPA.^12^ The goal of therapy is to achieve symptom resolution, clearance of radiographic infiltrates, and establishment of a stable baseline serum level of total IgE. During the acute episode, usual starting dose of prednisone is 0.5 mg/kg daily for two weeks, which is then reduced to an alternate day regime for 2–3 months, and finally tapered off over next 2–3 months.^13^–^14^ In patients with recurrent flares of ABPA, or in those with severe persistent asthma, long-term corticosteroid therapy may be necessary to control symptoms. Patients in fibrotic stage of ABPA may have increased sputum volume as a result of infection. Measures such as postural drainage and antibiotics may be useful, but with progression of disease, exercise tolerance decreases and oxygen therapy may be needed. Optimization of baseline asthma therapy is essential with inhaled corticosteroid and β_2_-agonists. Itraconazole in doses of 200 mg/day for two years in ABPA is helpful in reducing the need of corticosteroid therapy, along with clinical, biological, and functional improvements.^15^
